# Pedalitin as an Interesting Phytocompound with a High Potential for Further Functionalization and Application in the Prevention and Treatment of Lifestyle Diseases—The First Narrative Review

**DOI:** 10.3390/antiox15070792

**Published:** 2026-06-25

**Authors:** Monika Stompor-Gorący, Robert Ostatek, Agata Bajek-Bil, Małgorzata Kus-Liśkiewicz, Agnieszka Gala-Błądzińska, Maciej Machaczka

**Affiliations:** 1 Department of Organic Chemistry, Faculty of Medicine, Collegium Medicum, University of Rzeszów, Rejtana 16C, 35-959 Rzeszów, Poland; 2 Department of Pathophysiology, Faculty of Medicine, Collegium Medicum, University of Rzeszów, Rejtana 16C, 35-959 Rzeszów, Poland; mmachaczka@ur.edu.pl; 3 Faculty of Chemistry, Rzeszów University of Technology, 35-959 Rzeszów, Poland; rostatek@prz.edu.pl (R.O.); abajek@prz.edu.pl (A.B.-B.); 4 Faculty of Biotechnology, Collegium Medicum, University of Rzeszow, Pigonia 1, 35-310 Rzeszow, Poland; mkus@ur.edu.pl; 5 Department of Nephrology and Endocrinology, Faculty of Medicine, University of Rzeszów, al. Tadeusza Rejtana 16C, 35-959 Rzeszów, Poland; agala@ur.edu.pl; 6 Clinic of Nephrology and Dialysis Unit, Fryderyk Chopin University Clinical Hospital, ul. Fryderyka Szopena 2, 35-055 Rzeszów, Poland; 7 Department of Clinical Science and Education, Division of Internal Medicine, Karolinska Institutet Södersjukhuset, 11883 Stockholm, Sweden

**Keywords:** pedalitin, pedalitin-6-*O*-glucoside, methoxylated flavones, pharmacological effects

## Abstract

Methoxyflavones, along with chalcones and phenolic acids, belong to natural polyphenols. Apart from their antioxidant function, in plants, they act as pigments and are a part of plant chemical defense system. A member of this group is pedalitin, which expresses a range of interesting biological properties. The compound acts as an anticancer agent, strengthens the immune system, improves insulin sensitivity, protects internal organs, and also shows anti-inflammatory and neuroprotective activity. Due to the constant need for alternative therapies for treatment and prevention of lifestyle diseases, *O*-methylated flavonoids, including pedalitin, may serve as natural lead molecules in designing new structures with significant pharmacological potential. This review summarizes the literature reports on obtaining pedalitin and assessing its biological activity, also in the context of further functionalization of this compound and potential new applications.

## 1. Introduction

**Figure 1:**
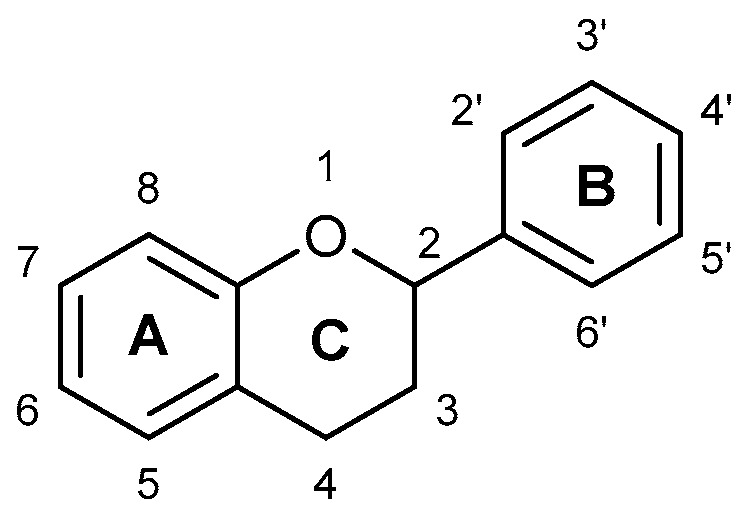
The basic chemical structure of flavonoids showing the standard carbon numbering system for rings A, B, and C. Variation in the B-ring attachment point and the oxidation/unsaturation state of the C-ring define the different subgroups.

Flavonoids are a group of natural compounds of plant origin that are taken in diets. Their structural diversity (Figure 1) determine both their bioavailability and pharmacological activity [1,2]. Under physiological conditions, they undergo glucuronidation, methylation, or sulfate conjugation in the intestinal mucous membrane. In further steps of the metabolism, they are decomposed in the colon to low-molecular-weight compounds [3]. It has been shown that flavonoids have beneficial effects on the cardiovascular system, osteoarticular system, and skin [4,5,6]. They may be also used in treatment of cancers and some metabolic diseases [7,8,9,10].

Chemical structures of flavonoids, i.e., the presence of methoxy and prenyl groups as well as the presence of hydroxyl groups in the aromatic rings A, B, and C, determine their physicochemical properties and have an impact on their reactivity, metabolic stability, bioavailability, and pharmacological activity [11,12,13,14]. Another significant aspect of biological activity of *O*-methylphenols is their structure-dependent capability to modulate receptors in the central nervous system, such as ionotropic GABA_A_ receptors or estrogen receptors [15,16]. These properties may be useful in a search for new drugs with 2-phenylchromane structure to be used, e.g., in the treatment of neurological diseases or in innovative hormone replacement therapies (HRT). The polyphenolic structures of flavonoids and isoflavonoids confer them also with the ability to scavenge free radicals and to chelate transition metals, being the basis for their potent antioxidant properties [17].

The chemical structures of phenolics determine also their interactions with the cell membranes [18], which are associated with biological effects exerted by flavonoids (e.g., protection from lipid oxidation or multidrug resistance modulation) [19]. Numerous scientific studies have shown that methylated flavonoids exhibit much better biological properties than their non-methylated analogues. In addition, the methylation of a flavonoid aglycone significantly increases its metabolic stability and enhances the membrane transport, resulting in easier absorption and greater bioavailability.

**Figure 2:**
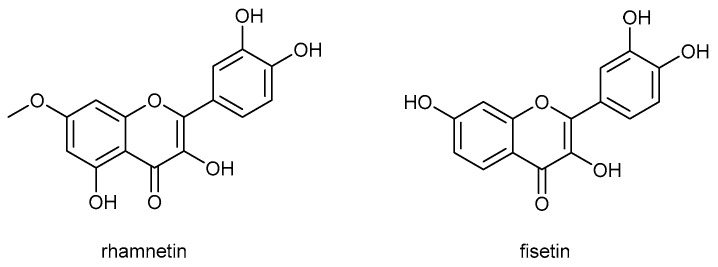
Examples of bioactive tetrahydroxyflavones.

7-Methoxytetrahydroxyflavones, such as pedalitin, are common plant secondary metabolites. Additionally, many bioactive pedalitin analogues are known. For instance, rhamnetin (Figure 2), found in several plant species, has a range of health-promoting activities [20,21]. There is also growing interest in natural dietary antioxidants, which have high anticancer activity, such as fisetin (3,3′,4′,7-tetrahydroxyflavone) [22]. What is interesting, fisetin may find application in the treatment of cognitive impairment related to diabetes [23]. Moreover, due to its strong antioxidant action, it may be an important factor for inhibiting biological aging [24].

Due to the broad spectrum of biological activities of natural *O*-methylated flavonoids, there is growing interest in new methods of obtaining of these compounds and improvement of their bioavailability. An interesting research topic is also synthesis of new molecules on the basis of the simple compounds isolated from natural sources, followed by determination of their properties, which is fundamental for development of medicinal chemistry and new medications. The objective of this review was to summarize scientific reports on pedalitin—*O*-methylated flavanol, which is commonly found in various plant species—in order to support the development of new methods of its functionalization based on confirmed and significant pharmacological activity. These may open new perspectives for the innovative research in this field as well as for applicational aspects in pharmaceutical chemistry.

**Scheme 1:**
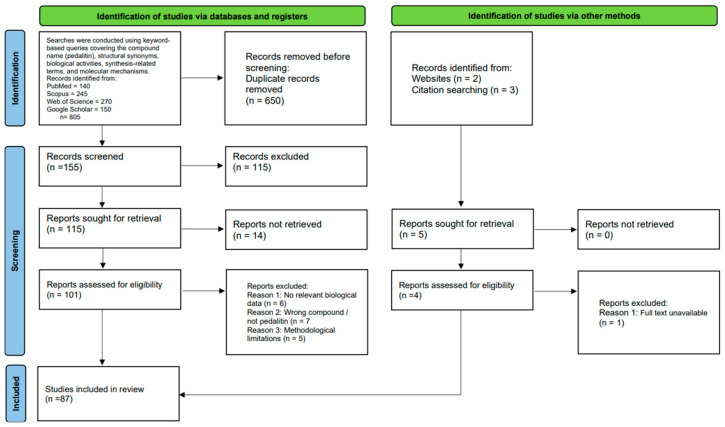
The PRISMA flow diagram of inclusion in the review.

A systematic search was performed to identify all relevant studies. We searched PubMed, Scopus, Web of Science, and Google Scholar. All databases were searched from their inception to November 2025. Boolean operators (and, or), truncation, and phrase searching were applied. The search terms included the following: pedalitin or “*O*-methylated flavonoid”, pedalitin and (anticancer or anti-inflammatory or neuroprotective or antioxidant), pedalitin and (isolation or extraction or synthesis), and (pedalitin) and (oxidative stress or ROS). Studies were eligible for inclusion if they met the following predefined criteria: investigated pedalitin or structurally related *O*-methylated flavonoids; reported biological, biochemical, pharmacological, or mechanistic outcomes relevant to lifestyle diseases; were original research articles presenting in vitro, in vivo, or in silico data; were published in English or German; and were available in full text. Studies were excluded if they were reviews, meta analyses, conference abstracts, editorials, or book chapters; did not examine pedalitin or did not report relevant biological outcomes; lacked sufficient methodological detail or extractable data; or were duplicates or retracted publications. The flow diagram of the study selection process following the PRISMA guidelines is presented in Scheme 1.

## 2. Chemical Characteristics of Pedalitin, Its Derivatives, and Their Sources

### Structure and Chemistry of Pedalitin

**Figure 3:**
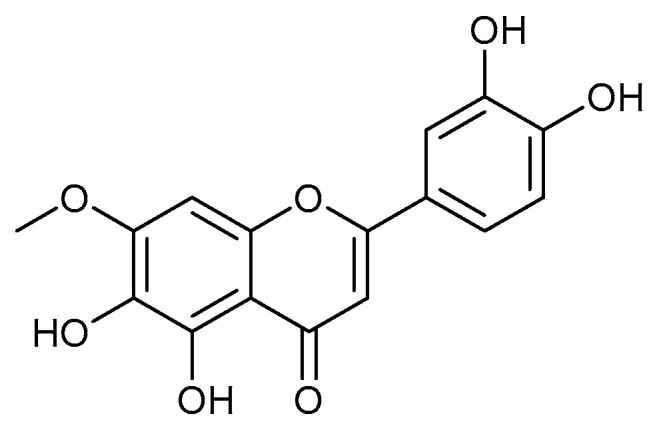
Chemical structure of pedalitin.

In terms of the chemical structure, pedalitin belongs to polyphenolic compounds with the 2-phenylchromen-4-one (2-phenyl-1-benzopyran-4-one) skeleton. It contains one methoxy group and four hydroxyl ones attached to the rings A and B (Figure 3). From a chemical point of view, it is a tetrahydroxymonomethoxyflavone, with four hydroxyl groups at C-3′,-4′, 5, and 6 and one methoxy group at C-7.

**Figure 4:**
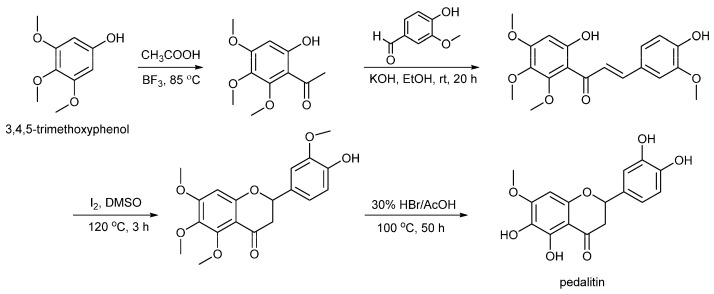
Chemical synthesis of pedalitin.

Because of the important biological activity of pedalitin, specifically its function as a tyrosinase and alpha-glucosidase inhibitor, a method for its chemical synthesis has been developed. Although reports on this subject are limited, it can be synthesized from cheap and readily available precursors, namely 3,4,5-trimethoxyphenol and vanillin (Figure 4) [25], although a few other approaches requiring many chemical transformations were reported earlier [26,27]. In a short reaction sequence, involving aldol condensation, followed by intramolecular oxidative cyclisation of the intermediate chalcone, catalyzed by iodine, and finally, demethylation of 5, 6, and 3′ methoxy groups, pedalitin was obtained in 35.3% yield, with the spectral data as follows: ^1^H NMR (500 MHz, DMSO-d_6_) δ 12.6 (s, 1H), 9.97 (s, 1H), 9.38 (s, 1H), 8.73 (s, 1H), 7.45 (s, 2H), 6.92–6.87 (m, 2H), 6.71 (s, 1H), 3.93 (s, 3H); ^13^C NMR (125 MHz, DMSO-d_6_) δ 182.5, 164.4, 154.7, 150.08, 150.06, 146.70, 146.23, 130.8, 122.2, 119.3, 116.4, 113.9, 105.4, 103.0, 91.4, 56.7; ESI-HRMS m/z [M + Na]^+^ calcd for C_16_H_12_NaO_7_ 339.0481; found, 339.0479.

**Figure 5:**
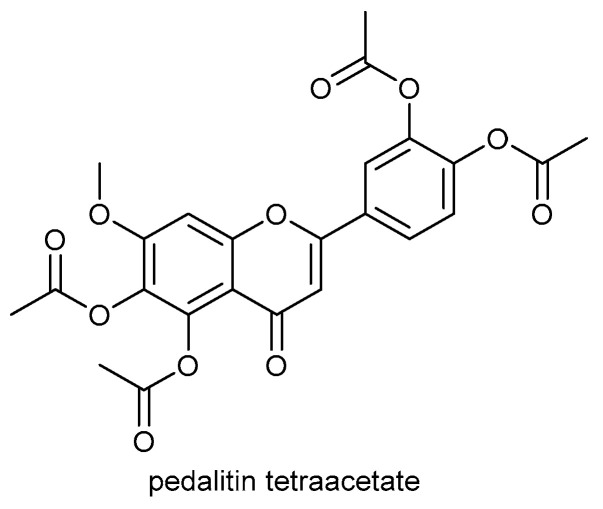
Chemical structure of pedalitin tetraacetate.

Moreover, the crystal structure of a pedalitin derivative in the form of pedalitin tetraacetate (Figure 5) was determined by X-ray crystallography.

## 3. Sources of Pedalitin and Its Derivatives

**Figure 6:**
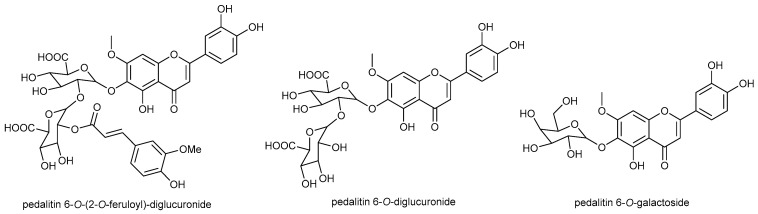
Chemical structures of pedalitin derivatives found in plants.

In plants, pedalitin can be found in the form of aglycone and also as the glucosides and glucuronides. Pedalitin 6-*O*-diglucuronide, pedalitin 6-*O*-(2-*O*-feruloyl)-diglucuronide, and pedalitin 6-galactoside (Figure 6) were identified, among others, in hydroalcoholic extracts from *Verbena officinalis* L. by means of chromatographic methods HPLC-DAD and LC–MS [28].

**Figure 7:**
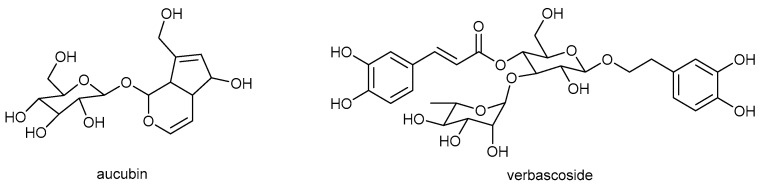
Chemical structures of some iridoid and phenylpropanoid glycosides.

For comparison, the main groups of secondary metabolites that determine the profile of biological activity of verbena comprise iridoid glycosides, e.g., verbenalin (verbanaloside), aucubin (Figure 7), and hastatoside, and also phenylpropanoid glycosides (caffeic acid derivatives), such as verbascoside (acteoside), isoverbascoside (isoacteoside), and eukovoside, the content of which depends on the plant growing season [29].

Due to high content of polyphenols, antioxidant properties, and a wide range of pharmacological activities of common vervain herb (*Verbena officinalis* L.), this plant has become an ingredient of botanical dietary supplements [30]. Stationary cultures of vervain herb, as well as agitated and bioreactor ones, are the reach sources of bioactive phenolic compounds for pharmacy, healthy food production, and cosmetology; therefore, there is research conducted on optimalization of their biosynthesis, aimed to achieve an overproduction of the most desired bioactive compounds [31]. In addition, innovative delivery systems of active compounds from verbena in the form of Au/CuO/ZnO nanoparticles have been being developed, and their strong cytotoxic activity against cancer cells was confirmed using Jurkat cell line [32].

Moreover, pedalitin can be obtained usually by extraction of the whole plants or their individual parts with organic solvents such as methanol, ethanol, or ethyl acetate followed by preparative liquid chromatography or analytical chromatography from various medicinal herbs and leguminous plants, including *Pterogyne nitens* [33], *Pedalium murex* L. [34,35], *Rabdosia serra* [36], *Halophila johnsonii* [37], flowers of *Sesamum indicum* [38], young sesame leaves of *Sesamum indicum* [39], *Eremosparton songoricum* [40], *Isodon enanderianus* [41], Ratibida latipalearis [42], leaves of *Isodon rubescens var lushiensis* [43], *Eupatorium inulaefolium* [44], several species of *Sullivantia* (*S. oregana, S. purpusii, S. hapemanii, S. halmicola, S. renifolia, S. sullivantii*) [45], *Rabdosia japonica* [46], *Isodon excisoides* [47], *Callicarpa nudiflora* Hook [48], fresh flowers of *Artemisia scoparia* [49], *Teucrium hyrcanicum* [50], leaves of *Penstemon frutescens* [51], and *Anisomeles ovata* [52]. Apart from that, pedalitin has been isolated from *Ruellia tuberosa* [53].

The extraction and recovery of the pedalitin depend not only on the isolation method applied, but also on the plant species and even the specific part from which the substance is extracted. Pedalitin was isolated by methanolic extraction of dried leaves of *Plectranthus ornatus* or aerial parts of *Salvia circinate*, the plants known for their medicinal applications in the treatment of gastrointestinal, skin, and genitourinary diseases, with various yield of extraction of 17 mg/4.2 kg or 20 mg/400g dry and powdered plant material, respectively [54,55].

According to the most recent reports, pedalitin and its derivatives can be also isolated by microemulsion capillary electrophoresis (MECE) from *Isodon japonica* [56]. Using a borax-based buffer, baseline separation was achieved in under 10 min, yielding a pedalitin limit of detection (LOD) of 0.82 µg/mL.

**Figure 8:**
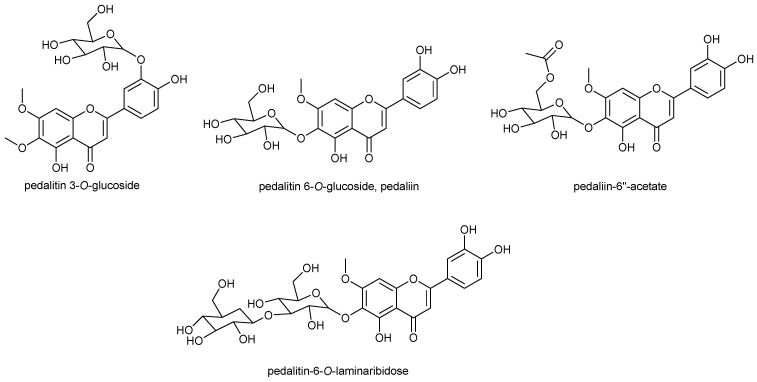
Chemical structures of pedalitin derivatives with glucose unit.

Pedalitin, except for the aglycone, in plants is most often found linked to the glucose unit, namely pedalitin 6-*O*-glucoside, known also as pedaliin [57]. Glycoside derivatives (Figure 8) of pedalitin: pedalitin-3′-*O*-β-glucoside (20 mg) and pedalitin-6-*O*-glucoside (36 mg) as well as pedaliin-6″-acetate (16 mg) were also isolated and characterized from air-dried whole plants of *Dracocephalum tanguticum* (5 kg) [58]. Moreover, Fuji et al. [39] reported isolation of pedalitin-6-*O*-laminaribioside as a yellowish amorphous powder besides pedalitin and pedaliin from young sesame leaves of *Sesamum indicum*.

Due to the broad spectrum of biological activity and potential industrial applications, methoxylated flavonoids, including pedalitin, can be also obtained by modern biotechnological methods, which is of interest to the nutraceutical industry. Berim and Gang [59] proposed the method using the set of enzymes from sweet basil (*Ocimum basilicum*) to construct five strains of *Saccharomyces cerevisiae* producing 8- and/or 6-substituted, methoxylated flavones from their natural precursor apigenin.

## 4. Biological Properties of Pedalitin

### 4.1. Antioxidant Activity

An antioxidant activity of flavonoids is related to various mechanisms. The direct way of action is to reduce reactive oxygen species generation in cells by inhibiting the activity of the enzymes involved in this process, such as xanthine oxidase, NAD(P)H oxidase or myeloperoxidase.

Fernandes et al. [60] isolated from *Pterogyne nitens* (Fabaceae) pedalitin and pedalitin 6-*O*-*β*-glucopyranoside, which were next evaluated for their effect on enzymatic activity of myeloperoxidase (MPO). The most active was pedalitin with the IC_50_ of 3.75 nM, whereas the IC_50_ of the 6-*O*-*β*-glucopyranoside was 4-fold higher (15.8 nM). Assessment of the antioxidant activity by ABTS and DPPH methods confirmed higher activity of pedalitin than of its glucoside and of quercetin, used for comparison (ABTS assay). The IC_50_ determined for pedalitin in both tests were 1.4 µM and 5.2 µM, respectively.

Isolated from the dried leaves of *Rabdosia japonica* Hara (Labiatae), pedalitin exhibited superoxide radical scavenging activity and the inhibitory effect on xanthine oxidase (EC 1.1.3.22). The compound prevents the generation of superoxide radicals in part by inhibiting xanthine oxidase competitively (IC_50_ = 3.7 µM). Moreover, pedalitin inhibited uric acid formation by xanthine oxidase (IC_50_ = 18.2 µM), and the inhibition kinetics analyzed by Lineweaver-Burk plots found pedalitin to be the competitive inhibitor [61].

Pedalitin is also one of the main ingredients of the ethyl acetate extract obtained from aerial parts of *Graziella gaudichaudeana*, known for its high antioxidant activity, with an IC_50_ value of 13.88 ± 0.04 μg mL^−1^ [62]. Pedalitin isolated from *Eremosparton songoricum* (Litv) demonstrated the ability to inhibit peroxidation of lipids, with an IC_50_ of 28.6 μM [63].

The capability of flavonoids to scavenge ROS and chelate transition metals may be important for the treatment of pathological conditions associated with oxidative stress (e.g., inflammation, circulatory system disorders, diabetes, neurodegenerative diseases, or cancers). Because pedalitin is found in plants also in the form of glycosides, its derivatives were also examined for possible pro-health activity. It was proven that pedalitin-6-*O*-glucoside, which is the main ingredient of sesame leaf extract (*Sesamum indicum* L.), has antioxidant and antitumor activity towards colon cancer cells (HCT 116) [64]. It was shown that thermal treatment of ethanolic extracts of this plant (*Sesamum indicum* L.) has no influence on the content of both pedalitin and pedaliin. The extracts demonstrated antioxidant properties and inhibitory effects against growth and metastasis of human colon cancer cells HT29 and HCT 116 [65].

The mentioned earlier derivatives of pedalitin and pedaliin, pedalitin-6-*O*-glucoside, pedalitin-3′-*O*-β-glucoside and pedaliin-6″-acetate, were isolated from whole plants of *Dracocephalum tanguticum* by extraction with ethanol 95%. The compounds displayed various antioxidant activity, determined using the 1,1-diphenyl-2-picrylhydrazyl (DPPH), ferrous ions, and ABTS radical cation radical in vitro assay [58].

Okumura et al. [66] developed a fast method for screening the antioxidant activity of pedalitin found in *Pterogyne nitens*, using electrochemical methods.

Due to its antioxidant properties, new pharmaceutical and cosmetic formulations containing pedalitin were developed in the form of cytocompatible hydrogels, which can be used for healing wounds [67].

### 4.2. Anti-Diabetic Properties

As we know, the pathogenesis of diabetes is related either to impaired production and secretion of insulin or resistance of target tissues to this hormone, which leads to raised blood glucose levels. Experimental data showed that some flavonoids have anti-diabetic properties [68,69].

It has been reported that pedalitin found in the extract of *Salvia circinata* is a natural α-glucosidase inhibitor. It reduced significantly the postprandial peak during an oral sucrose tolerance test in healthy mice [70]. In addition, pedalitin exhibits great inhibitory effects against tyrosinase and α-glucosidase [71]. Pedalitin showed a very strong inhibitory effect, with IC_50_ value of 0.28 mM, on mushroom tyrosinase. The IC_50_ value calculated for the inhibitory effect of pedalitin on yeast α-glucosidase was 0.29 mM.

It has been also reported that pedalitin, as well as infusions prepared from the aerial parts of the plant *Salvia amarissima* Ortega, have inhibitory activity towards tyrosine phosphatase 1B (PTP-1B)—the enzyme where overexpression contributes to the development of insulin resistance and metabolic disorders (obesity and diabetes) [72]. These data were supported by in silico studies, where computational methods, including molecular docking, binding free energy estimates, and ADMET predictions, were performed. It is reported that pedatilin exhibits significant potential as an anti-diabetic agent through the multi-target inhibition of aldose reductase, DPP-4, and α-glucosidase [73]. To summarize the aforementioned reports, this chemical should be developed further into novel pharmaceuticals for treating diabetes mellitus.

### 4.3. Antiviral and Antimicrobial Activity

Pedalitin from *Pterogyne nitens* inhibited infection with Hepatitis C virus by blocking HCV entry to cells [33] It was found that pedalitin can inhibit virus entry up to 80% by the direct interaction with the virus particles and up to 72.2% by the interactions with the host cells. Moreover, in the cell-based assays pedalitin demonstrated significant activity against Zika virus (ZIKV) at the concentrations of 250 and 500 µM [74].

Sangalli-Leite et al. [75] demonstrated antifungal activity of pedalitin administered in combination with amphotericin B against *Cryptococcus* sp., the pathogen responsible for development of opportunistic fungal infections, which cause high morbidity and mortality in patients with weakened immune systems. In the in vitro assay, the IC_50_ for pedalitin was found to be 3.9 mg/L, while in the combination treatment with amphotericin B, it decreased to 1.0 mg/L. The combination of two antifungal substances was more effective than monotherapy, which was also confirmed using in vivo mouse models.

Although pedalitin isolated from *Arctotis arctotoides* shows no growth inhibitory activity against common bacterial strains such as *Bacillus subtilis*, *Staphylococcus aureus*, *Escherichia coli*, and *Shigella sonnei* (MIC greater than 250 µg/mL^−1^) [76], it may still hold niche value. Specifically, it has demonstrated targeted antibacterial activity against *Helicobacter pylori*, a globally prevalent gastrointestinal pathogen [77]. Therefore, while not a broad-spectrum antibiotic candidate, it warrants further investigation for specialized narrow-spectrum applications.

### 4.4. Hepaptoprotective Activity

Fraga et al. [78] studied protective properties of pedalitin to liver cells against the injury induced by the oxidative stress generated by CCl_4_. The effect of pedalitin on *tert*-butyl hydroperoxide (*t*-BOOH)-initiated chemiluminescence of mouse liver homogenates was determined with an IC_50_ value of 200 µM. Other omics-based studies, which integrated data regarding the pedalitin content in black sesame, demonstrate that thermally processed black sesame pigment (FBSP) acts as a powerful functional agent. It protects the liver by repairing the gut microbiome and regulating the HIF-1 pathway, making it a highly promising candidate for functional foods targeting fatty liver diseases [79].

### 4.5. Anti-Inflammatory Properties

An in vitro study on a cell model confirmed that pedalitin significantly reduced triacylglycerols (TG) level and lipid formation. Additionally, it downregulated the expression levels of key factors in fatty acid metabolism (CPT2, HADH), inflammatory factors (IL-17, TNF-α), and the FOXO signaling pathway (EGFR, IRS1, AKT1, FOXO1) in LO2 cells treated with this compound [80].

Pedalitin, isolated from the aerial part of *Rabdosia japonica* (Labiatae), inhibited soybean lipoxygenase-1 (EC 1.13.11.12, Type I) with an IC_50_ value of 152.5 µM [79]. Earlier reports documented inhibitory activity of flavones with vicinal hydroxyl groups in the aromatic ring B, i.e., pedalitin and cirsiliol, toward 5-lipoxygenase, the enzyme that initiates biosynthesis of leukotrienes. Pedalitin, with the 6-hydroxyl group was as active as cirsiliol, which has a 6-methoxy group. Demethylation at the 7 position reduced the inhibitory effect [81].

Pedalitin isolated from *Anisomeles indica* inhibited the overproduction of inflammatory mediators, i.e., NO, TNF- α (*tumor necrosis factor-alfa*), and IL-12, by LPS/IFN-γ stimulated macrophages at a concentration of 40 µM. At a concentration of 20 µM, it significantly arrested the cell cycle in spleen cells stimulated by concanavalin A [82]. Moharram et al. [83] identified pedalitin in the leaves of *Salvia* splendens Sellow ex Roem. The plant extract containing a part from pedalitin, a range of biologically active compounds, including caffeic acid, rosmarinic acid, and its derivatives, luteolin, apigenin and citronellol, demonstrated considerable hypoglycemic, anti-inflammatory, and antioxidant effects. These reports are consistent with other in silico analyses of pedalitin, in which molecular docking studies demonstrated its ability to inhibit PLA2 and p38 MAPK, both of which play critical roles in the production of inflammatory cytokines [84].

### 4.6. Analgesic and Anxiolytic Properties

According to Moreno-Pèrez et al. [55], pedalitin isolated from the methanolic extract of *Salvia circinata* enhanced the antinociceptive effect of the extract in alleviating abdominal pain in vivo. Similarly, methanolic extract of *Anisomeles indica*, containing pedalitin, along with other active substances, such as 3,4-dihydroxybenzoic acid, betonyoside A, isoacteoside, and terniflorin, demonstrated considerable antinociceptive, anxiolytic, and sedative activity; therefore, it may be used in the treatment of neuropharmacological abnormalities. The authors demonstrated significant antinociceptive, anxiolytic, and sedative activities in experimental mouse models. Although 3,4-dihydroxybenzoic acid showed the strongest predicted activity among the analyzed compounds, pedalitin was highlighted as one of the bioactive flavonoids potentially involved in modulation of pain and inflammation-related pathways. The study further suggested that flavonoids present in *A. indica* may exert their effects through interactions with COX-1, COX-2, serotonergic targets, and inflammatory mediators [85,86].

### 4.7. Pedalitin and Cancers

Because epidemiological observations indicate an inverse correlation between an intake of flavonoids by humans and the risk of some types of cancers, an important research area is to assess the effect of flavonoids on cancer cells. The research is meaningful also due to the fact that some flavonoids are being introduced to clinical practice, like, for example, quercetin, epigallocatechin-3-gallate (phase I clinical trial), and flavopiridol (phase II clinical trial).

According to the reports published so far, pedalitin isolated from *Rabdosia rubescens* inhibited the growth of human cancer cells HepG2, COLO 205 and MCF-7 with an IC_50_ value of over 100 µM in a 24 h test, while the IC_50_ value determined for HL-60 was 61.98 µM. Pedalitin isolated from *Rabdosia japonica* was cytotoxic to B16-F10 melanoma cells with an IC_50_ = 30 µM [87].

Because the anticancer activity of flavonoids results not only from their antioxidant and anti-inflammatory properties but also from their influence on the enzymes responsible for the metabolism of xenobiotics, DNA replication, cell cycle regulation, and, indirectly, the modulation of cell metabolism, a detailed study on pedalitin within this area would deliver valuable information. It was confirmed that in cancer cell lines that are resistant to chemotherapeutics, flavonoids increase the concentrations of some commonly used cytostatics [88,89]. To the best of our knowledge, a similar study for pedalitin has not been described so far.

Pedalitin inhibited the chymotrypsin-like, caspase-like, or trypsin-like activity of 26S proteasome when Suc-LLVY-AMC, Z-LLE-AMC, and Ac-RLR-AMC were used as substrates. These results may be useful for the development of new methods of cancer treatment based on proteasome inhibition [90].

### 4.8. Other Significant Properties of Pedalitin and Its Natural Derivatives

Natural polyphenolic compounds have beneficial effects on the biosynthesis of collagen, elastin, and other ingredients of the extracellular matrix, improving the stability of the connective tissue. Pedalitin isolated from *Cirsium palustre* was tested for its influence on collagen expression in human skin fibroblasts. The results revealed that in the concentrations of 1, 20, and 40 µM, pedalitin increased the level of total collagen. In addition, it stimulates type I and III collagen expression and has inhibitory effect on the activity of matrix metalloproteinase-2 (MMP-2) [91]. Moreover, there are literature reports that pedalitin belongs to strong inhibitors of human protein kinase CK2 [92].

### 4.9. Clinical Summary

**Figure 9:**
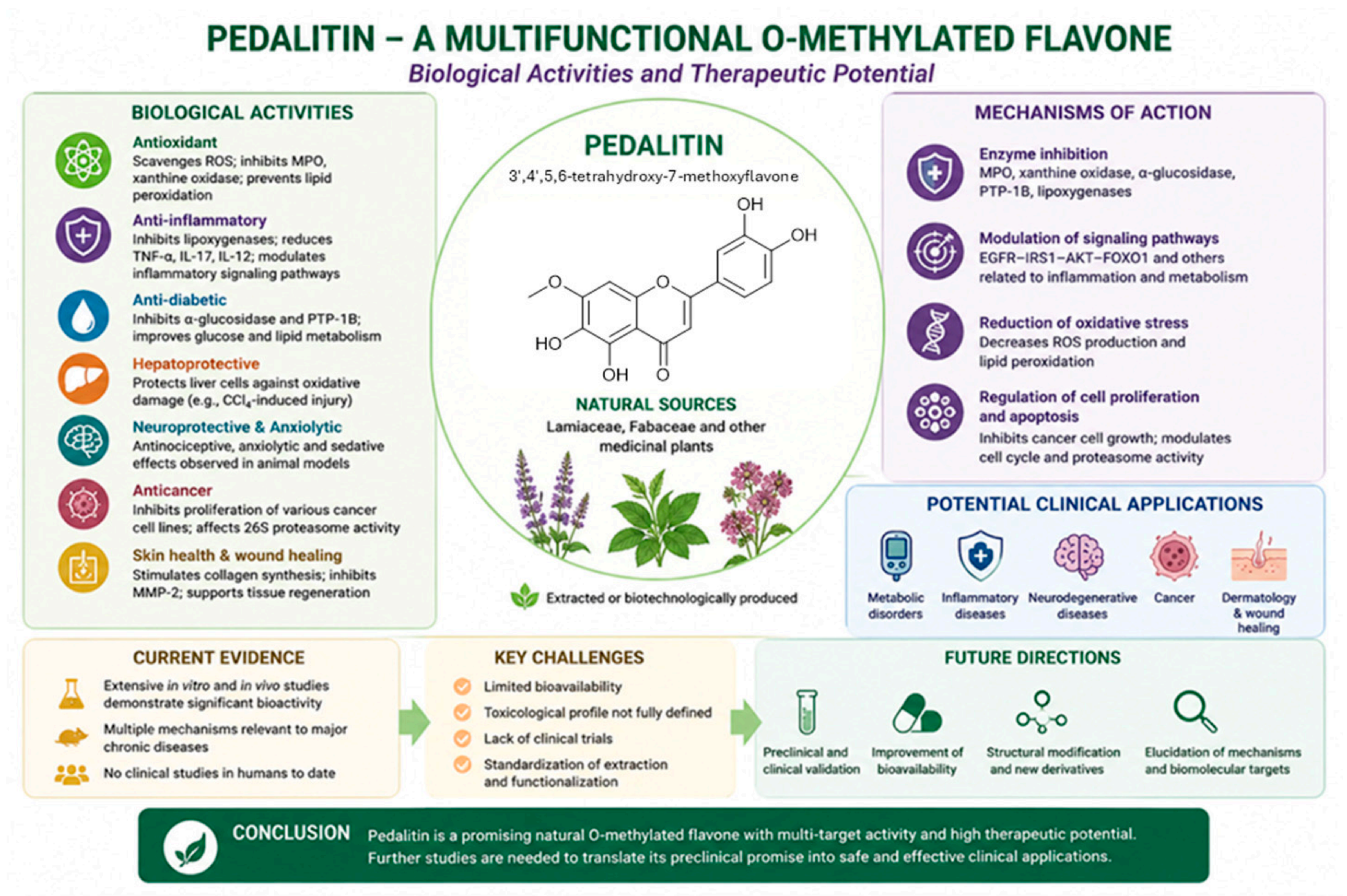
Integrated multifunctional profile of pedalitin.

From a clinical perspective, pedalitin represents a promising natural compound with pleiotropic biological activity, including antioxidant, anti-inflammatory, antidiabetic, hepatoprotective, and anticancer effects (Figure 9).

Experimental studies to date indicate its ability to modulate key enzymes and signaling pathways associated with oxidative stress, glucose and lipid metabolism, and cancer cell proliferation. Particularly noteworthy are its inhibitory effects on myeloperoxidase, xanthine oxidase, α-glucosidase, and inflammatory mediators such as TNF-α, IL-17, and IL-12. Despite promising preclinical findings, clinical studies confirming the efficacy and safety of pedalitin in humans are still lacking. Nevertheless, this compound may serve as a potential lead molecule for the development of novel therapies targeting metabolic, inflammatory, and neoplastic diseases.

## 5. Conclusions

Despite continuous progress in medicine, research on new therapeutic compounds is still ongoing because many currently available therapies remain ineffective in a significant proportion of patients and are often associated with severe adverse side effects. Consequently, there is a growing need to develop alternative or complementary treatment strategies, including these based on naturally occurring organic compounds with promising biological activities and improved safety profiles.

To address these unmet needs, we summarized the most recent research on pedalitin, as an interesting compound for further examination in the area of phytopharmacology. This review presents the current knowledge on the biological activity of this polymethoxylated metabolite so as to support further study on its possible therapeutic usage. We hope that it will open new possibilities in the areas of medicinal chemistry and phytopharmacology in the future.

## 6. Future Directions

Further research on pedalitin should focus on preclinical verification of its activity in vitro and in vivo, with special attention to its influence on biology of cancers derived from various organs, the modulation of signaling pathways, biophysical interactions with cell membranes, and toxicological tests, in order to find safe ways of administering pedalitin in vivo. The research should also include multidirectional functionalization of pedalitin, aiming to obtain its new analogues, together with an assessment of their biological potential with the help of various techniques and research models. Finally, new ways of improving the bioavailability of pedalitin should be indicated so as to make full profit of its therapeutic potential.

## Data Availability

No new data were created or analyzed in this study.

## Abbreviations

ABTS	2,2′-azino-bis(3-ethylbenzothiazoline-6-sulfonic acid
Ac-RLR-AMC	Ac-Arg-Leu-Arg-AMC, fluorogenic substrate for the 26S proteasome
ADMET	absorption, distribution, metabolism, excretion, and toxicity
AKT1	AKT serine/threonine kinase
B16-F10	murine melanoma cell line
COLO 205	human epithelial cell line
COX-1	cyclooxygenase-1
COX-2	cyclooxygenase-2
CPT2	carnitine palmitoyltransferase II
DPP-4	dipeptydylopeptydaza 4
DPPH	1,1-diphenyl-2-picrylhydrazyl
GABA_A_	gamma-aminobutyric acid
EGFR	estimated glomerular filtration rate
FOXO	O of forkhead box transcription factors
HADH	hydroxyacyl-CoA dehydrogenase
HCT-116	colon cancer cell line
HepG2	human liver cancer cell line
HL-60	human leukemia cell line
HPLC-DAD	high-performance liquid chromatography with diode-array detection
HRT	hormone replacement therapies
p38-MAPK	p38 mitogen-activated protein kinase mitogen-activated protein kinase
MCF-7	breast cancer cell line
MIC	minimum inhibitory concentration
MPO	myeloperoxidase
MMP-2	matrix metalloproteinase-2
LC-MS	liquid chromatography-mass spectrometry
LO2	human fetal hepatocyte line
TNF-α	tumor necrosis factor-alfa
PLA2	phospholipase A2
PTP-1B	towards tyrosine phosphatase 1B
Suc-LLVY-AMC	Suc-Leu-Leu-Val-Tyr-AMC, membrane-permeable calpain-specific fluorogenic substrate
Z-LLE-AMC	Z-Leu-Leu-Glu-AMC, peptide-AMC linked substrate used to measure the postacidic-like hydrolysing activity of proteasome
